# NMR based serum metabolomics reveals a distinctive signature in patients with Lupus Nephritis

**DOI:** 10.1038/srep35309

**Published:** 2016-10-14

**Authors:** Anupam Guleria, Avadhesh Pratap, Durgesh Dubey, Atul Rawat, Smriti Chaurasia, Edavalath Sukesh, Sanat Phatak, Sajal Ajmani, Umesh Kumar, Chunni Lal Khetrapal, Paul Bacon, Ramnath Misra, Dinesh Kumar

**Affiliations:** 1 Centre of Biomedical Research, SGPGIMS Campus, Lucknow-226014, India; 2 Department of Immunology, SGPGIMS Campus, Lucknow-226014, India; 3 Department of Biotechnology, Babasaheb Bhimrao Ambedkar University, Lucknow-226025, India; 4 Rheumatology Research Group, Division of Immunity and Infection, Birmingham University, UK

## Abstract

Management of patient with Lupus Nephritis (LN) continues to remain a challenge for the treating physicians because of considerable morbidity and even mortality. The search of biomarkers in serum and urine is a focus of researchers to unravel new targets for therapy. In the present study, the utility of NMR-based serum metabolomics has been evaluated for the first time in discriminating LN patients from non-nephritis lupus patients (SLE) and further to get new insights into the underlying disease processes for better clinical management. Metabolic profiling of sera obtained from 22 SLE patients, 40 LN patients and 30 healthy controls (HC) were performed using high resolution 1D ^1^H-CPMG and diffusion edited NMR spectra to identify the potential molecular biomarkers. Using multivariate analysis, we could distinguish SLE and LN patients from HC and LN from SLE patients. Compared to SLE patients, the LN patients had increased serum levels of lipid metabolites (including LDL/VLDL lipoproteins), creatinine and decreased levels of acetate. Our results revealed that metabolic markers especially lipids and acetate derived from NMR spectroscopy has high sensitivity and specificity to distinguish LN among SLE patients and has the potential to be a useful adjunctive tool in diagnosis and clinical management of LN.

Systemic lupus erythematosus (SLE) is a complex autoimmune disease characterized by diverse clinical manifestations which affect multiple end organs including joints, skin, heart, lungs, blood vessels and kidneys^1^,^2^. The most severe manifestation of SLE is renal involvement, the condition known as lupus nephritis (LN)^3^. About 50–60% of patients with SLE have nephritis with combinations of oedema, proteinuria, hypertension, urinary sediment abnormalities, hypocomplementemia and impaired renal function^4^,^5^,^6^. It may develop early in the course of SLE^7^, but in about 5–10% of cases, it becomes clinically apparent several years after the onset of SLE^5^,^6^,^8^. Despite advances in effective immunosuppressive therapies, the treatment of LN remains a challenge with considerable morbidity and progressive end stage renal disease requiring renal replacement therapy^1^,^3^. Renal biopsy is the gold standard for documenting histological class of nephritis and ascribing activity and damage features to guide the treatment^9^. However, being invasive, there are limitations on performing it serially for monitoring patients with LN. Thus there is an unmet need of biomarkers specific to nephritis among patients with SLE.

Metabolomics, the analysis of concentration profiles of low molecular weight metabolites present in biological fluids, has immense potential in identifying new biomarkers that are highly discriminatory for biological perturbations or diseased states^10^,^11^,^12^,^13^. Nuclear Magnetic Resonance (NMR) spectroscopy and mass spectrometry (MS) are the most widely used analytical techniques for metabolomics studies; the former being preferred as it is rapid, requires minimal sample preparation and provides highly reproducible results. Previous metabolomics studies have shown the potential of metabolic profiling in the diagnosis of many inflammatory and rheumatic diseases such as ulcerative colitis, Crohn’s disease, inflammatory bowel disease, osteoarthritis and rheumatoid arthritis^12^,^14^,^15^,^16^,^17^,^18^,^19^,^20^,^21^,^22^. Metabolic disturbances associated with SLE have also been reported in urine and serum using NMR spectroscopy and LC/MS and GC/MS-based approaches^23^,^24^,^25^. However, serum metabolomics of LN has not been studied so far to identify the metabolic changes to differentiate it from SLE. The aim of this study was to explore whether NMR-derived serum metabolomics would reveal a distinctive signature of LN and thereby, suggest a proof-of-principle for the use of NMR based serum metabolomics in screening LN.

## Results

### ^1^H NMR spectroscopy

**Figure 1:**
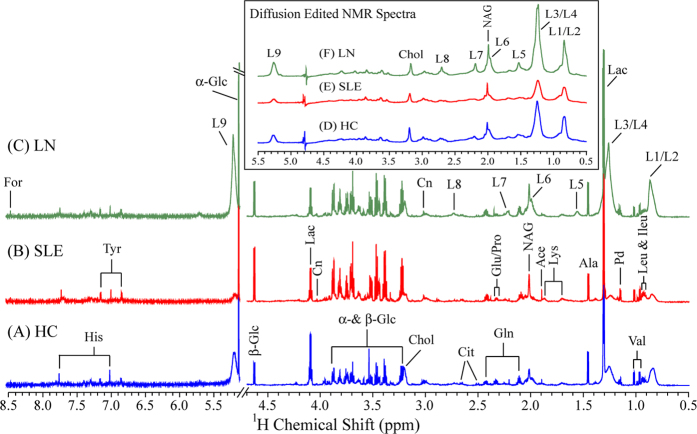
800-MHz ^1^H CPMG NMR spectra (δ0.5–4.7 and δ5.2–8.5) of serum obtained from the (**A**) HC, (**B**) SLE and (**C**) LN patient. Inset shows the diffusion edited NMR spectra (δ0.5–5.5) of serum obtained from the (**D**) HC, (E) SLE and (**F**) LN patient. The region of δ5.2–8.5 is magnified 8 times compared with the corresponding region of δ0.5–4.7 in the CPMG spectra for the purpose of clarity. Key: Ace: Acetate; Ala: Alanine; Cit: Citrate; Chol: Choline; Cn: Creatinine; For: Formate; Gln: Glutamine; Glu: Glutamate; His: Histidine; Ileu: Isoleucine; L: Lipid; L1/L2: CH_3_−(CH_2_)_n_− of LDL&VLDL; L3/L4: CH_3_−(CH_2_)_n_− of LDL&VLDL; L5: −CH_2_−CH_2_−C=O; L6: −CH_2_−CH=CH−; L7: −CH_2_−C=O; L8: =CH−CH_2_−CH=; L9: −CH=CH−; Lac: Lactate; Leu: Leucine; Lys: Lysine; NAG: N-acetyl glycoprotein; Pd: 1,2-propanediol; Pro: Proline; Tyr: Tyrosine; Val: Valine; α-Glc: α-Glucose; β-Glc: β-Glucose. (here HC: Healthy control; SLE: Systemic lupus erythematosus without nephritis; and LN: Lupus nephritis).

Figure 1 shows the representative 1D ^1^H Carr Purcell Meiboom Gill (CPMG) NMR spectra of sera obtained from a HC, SLE and LN patient, respectively. The respective diffusion edited NMR spectra are shown in the inset of Fig. 1. The 1D ^1^H CPMG spectra of serum samples showed signals mainly from lipids/lipoproteins (e.g. low-density lipoprotein (LDL), very low density lipoprotein (VLDL), unsaturated fatty acids (UFAs) etc.) and amino acids (e.g. alanine, valine, lysine, leucine, isoleucine, histidine, tyrosine, glutamine, glutamate and proline etc.). Other metabolites such as glucose, lactate, acetate, creatinine, citrate, formate, 1,2-propanediol, choline and N-acetyl glycoproteins (NAG) were also assigned well. Visual inspection of the CPMG spectra revealed higher levels of glucose and lower levels of lactate in LN/SLE sera compared to HC. Comparison of CPMG and diffusion edited spectra revealed clear differences in their lipid and lipoprotein profiles too. While SLE serum samples have decreased levels of lipids and lipoproteins, LN serum samples show increased levels of lipids and lipoproteins compared to HC (see inset of Fig. 1).

### Multivariate analysis and discovery of potential biomarkers

**Figure 2:**
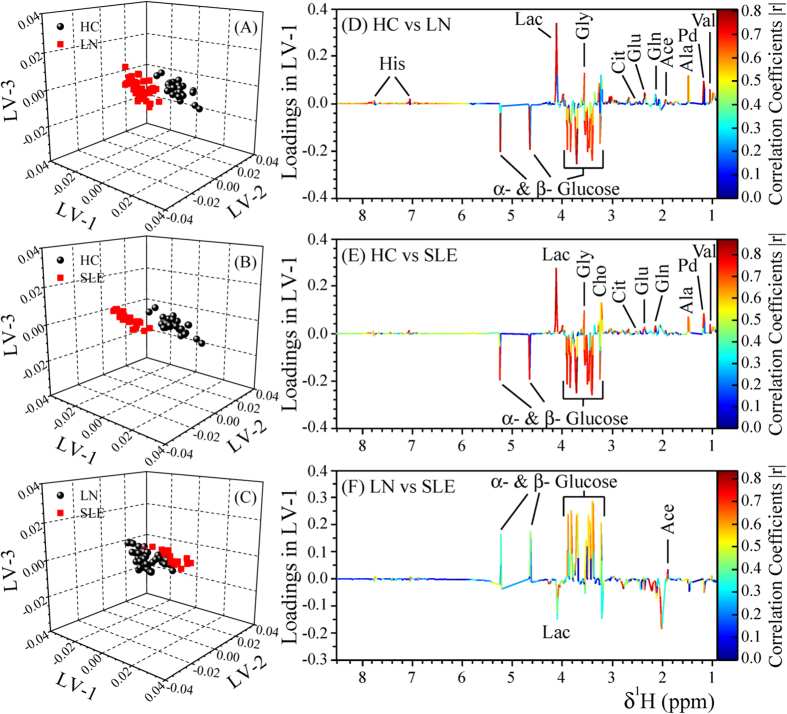
PLS-DA score plots derived from 1D CPMG ^1^H NMR spectra of serum samples (after the removal of lipid regions) between groups, (**A**) HC & LN, (**B**) HC & SLE, and (**C**) LN & SLE. (**D–F**) shows the color coded coefficient loading plot corresponding to the PLS-DA analysis shown in (**A–C**), respectively. The loading plots clearly demonstrate the metabolites responsible for the discrimination of the two groups in the corresponding score plots. The correlation coefficient of /r/>0.419 was used as the cut-off value for the statistical significance based on the discrimination significance at the level of p = 0.05. Peaks in the positive direction (>0) indicate the metabolites which are more abundant in the groups in the positive direction of first principal component. Consequently, metabolites which are more abundant in the groups in the negative direction (<0) of first principal component are presented as peaks in the negative direction. Notions used for the assignments are same as that were used previously in Fig. 1.

**Figure 3:**
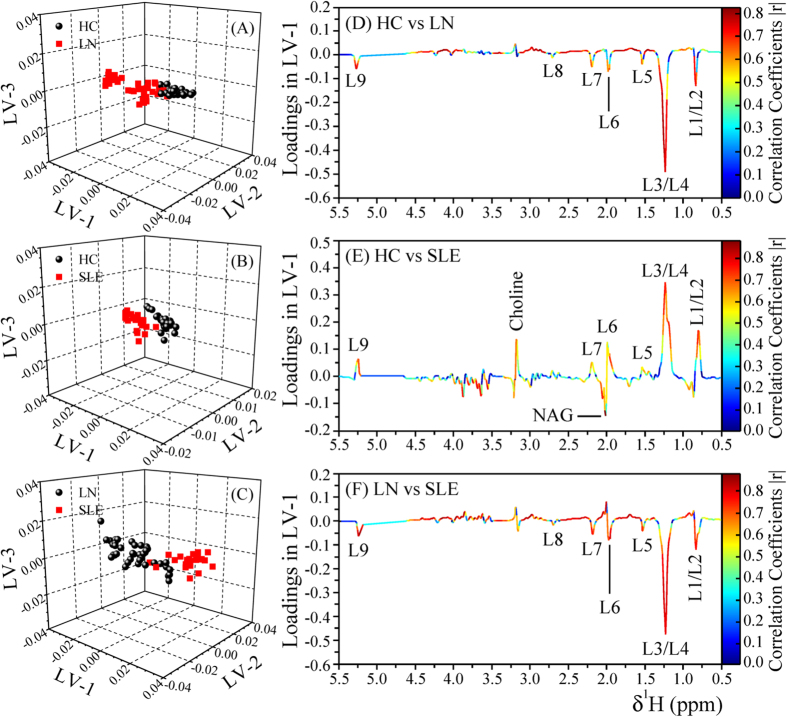
PLS-DA score plots derived from 1D diffusion edited ^1^H NMR spectra of serum samples between groups, (**A**) HC & LN, (**B**) HC & SLE, and (**C**) LN & SLE. (**D–F**) shows the color coded coefficient loading plot corresponding to the PLS-DA analysis shown in (**A–C**), respectively. The loading plots clearly demonstrate the metabolites responsible for the discrimination of the two groups in the corresponding score plots. The correlation coefficient of /r/>0.434 was used as the cut-off value for the statistical significance based on the discrimination significance at the level of p = 0.05. Peaks in the positive direction (>0) indicate the metabolites which are more abundant in the groups in the positive direction of first principal component. Consequently, metabolites which are more abundant in the groups in the negative direction (<0) of first principal component are presented as peaks in the negative direction. Notions used for the assignments are same as that were used previously in Fig. 1.

Principal Component Analysis (PCA) was performed on the NMR spectra and the obtained score plots showed a clear trend of group clustering and discrimination between the three cohorts (see supplementary Figures S1 and S2). One outlier was found in the healthy control group due to excessive alcohol peaks in its NMR spectrum and was excluded from the analysis. Pairwise Partial Least Squares Discriminant Analysis (PLS-DA) was further performed on both the CPMG and diffusion edited spectra. The PLS-DA score plots (Figs 2 and 3A–C), Supplementary Figure S3) showed that the clusters of LN and SLE patients are well separated from HC cluster and were also well discriminated from each other indicating that the metabolic profiles of LN and SLE patients were quite different. The model parameters for the explained variation, R^2^, which indicates goodness of fit and the predictive capability, Q^2^, were significantly higher (R^2^, Q^2^ > 0.5), indicating that the models possessed a satisfactory fit with good predictive power (Table 1). To evaluate the robustness of method, random permutation tests with 100 permutations were performed with derived PLS-DA models. The validation plots shown in Supplementary Figure S4A–F revealed the validity of original PLS-DA models, as both the permuted R^2^ and Q^2^ values on the left were significantly lower than the corresponding original points on right and the Q^2^ regression lines had negative intercept on y-axis. Furthermore, the calculation of analysis of variance CV-ANOVA validated the results, with highly significant p values (Table 1). Receiver operating characteristic (ROC) analysis performed on the Y-predicted values for each model gave the area under the curve greater than 0.99. It is important to mention here that the SLE and LN patients involved in this study were all on immunosuppressive medications including hydroxychloroquine (HCQ), Azathioprine (AZA), Cyclophosphamide, Mycophenolate and prednisolone (see Table 2). Each of these drugs may have its effect on metabolism; however, as the study involves heterogeneous patients in terms of medication, these effects have partly been randomized and minimized. To further rule out, if the medication has any profound effect on metabolism, the serum metabolic profiles of SLE/LN patients receiving HCQ/AZA medication were compared with those not receiving these medications using PLS-DA analysis (i.e. HCQ group vs non-HCQ group and AZA group vs non-AZA group). The resulted PLS-DA score plots (see Supplementary material, Figure S5) clearly revealed that there are no overt metabolic differences between SLE/LN patients receiving HCQ/AZA medication and not receiving this medication.

### Metabolic fingerprinting of LN and SLE patients

Metabolites responsible for discrimination in the PLS-DA score plots could be visualized by loading plots color-coded according to the absolute value of correlation coefficients (|r|), where a hot-colored signal (red) indicated more significant contribution to class separation than a cold-colored one (blue). Figures 2 and 3(D–F) illustrate the loading plots of metabolites in the first latent variable of PLS-DA between HC vs. LN, HC vs. SLE and LN vs. SLE, respectively, for low molecular weight metabolites and lipoproteins. The discriminatory metabolites for LN and SLE along with their chemical shifts, variable importance on projection (VIP) score and p-value are listed in Table 3. The serum of LN patients was characterized by lower levels of amino acids (such as leucine, valine, alanine, glutamate, proline, histidine and glycine), citrate, acetate, lactate and choline compared with HC, whereas levels of creatinine, lipoproteins (LDL and VLDL), Lipids (L5-L9), N-acetyl glycoprotein and α/β glucose were significantly elevated in sera of LN patients (Figs 2D and 3D). Compared with HC, the sera of SLE patients had lower levels of lipoproteins (LDL and VLDL), lipids, citrate, amino acids, lactate and choline, whereas levels of glucose and acetate were significantly elevated (Figs 2E and 3E). Consequently, the metabolites responsible for separating LN from SLE patients included elevated levels of lipoproteins (LDL and VLDL) and lipids, but decreased levels of acetate (Figs 2F and 3F).

**Figure 4:**
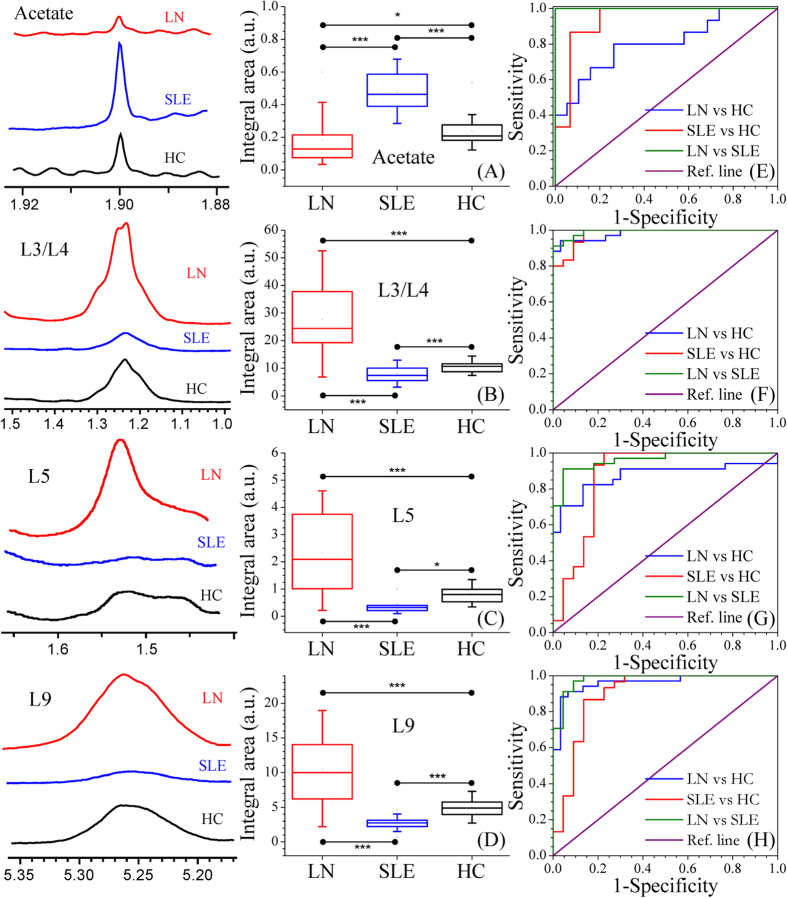
Representative box-cum-whisker plots showing quantitative variations of relative signal integrals for **(A)** Acetate, **(B)** L3/L4, **(C)** L5 and **(D)** L9 in LN, SLE and HC sera. (***p ≤ 0.001; *p ≤ 0.05). Red, blue and black bars represent LN, SLE and HC, respectively. In the box plots, the boxes denote interquartile ranges, horizontal line inside the box denote the median, and bottom and top boundaries of boxes are 25^th^ and 75^th^ percentiles, respectively. Lower and upper whiskers are 5^th^ and 95^th^ percentiles, respectively. Typical ^1^H NMR spectra of corresponding metabolites are shown in the left panel for all three groups. The potential of these metabolites in distinguishing LN from HC (blue dotted line), SLE from HC (red dotted line), or LN from SLE (green dotted line) were also analysed using the receiver’s operating characteristic (ROC) curves as displayed in **(E–H)**.

Further validation of the identified potential biomarkers was performed by quantifying and comparing the characteristic spectral regions of these metabolite entities in 1D ^1^H NMR spectra. PLS-DA was again repeated using the quantified metabolite data and comparable results to those as mentioned above were obtained (Supplementary Figure S6). The representative box plots of the relative signal integrals for the potential metabolic markers of LN such as acetate, LDL/VLDL and lipids are shown in Fig. 4(A–D). The relative signal integrals of the significant metabolites (median and range) have also been presented in Supplementary Table S1. Further, the specificity and selectivity of these potential biomarker metabolites were checked using ROC curves analysis. The resultant area under the ROC curve (AUROC) values are listed in Supplementary Table S2. The ROC curves for the potential biomarkers highlighted in panel A-D of Fig. 4 are also shown in Fig. 4(E–H), respectively. It is clear from the ROC analysis that lipids, lipoproteins and acetate with AUROC greater than 0.95 have the highest potential to be useful serum biomarkers for LN.

**Figure 5:**
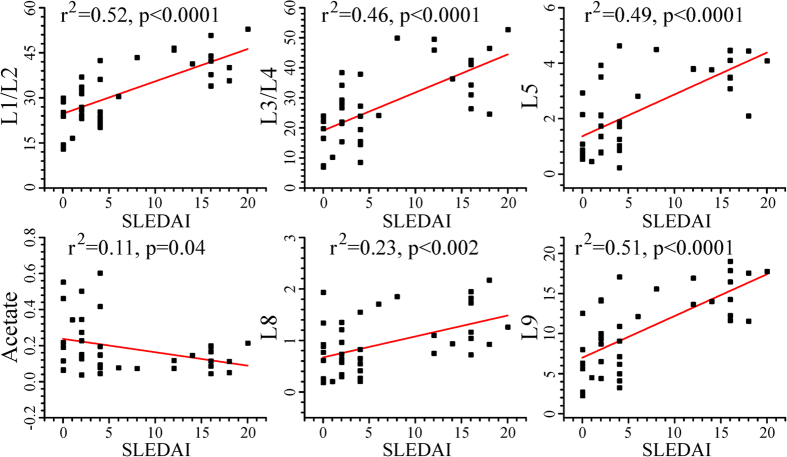
Serum levels of metabolic markers showing correlation with SLE disease activity index (SLEDAI) for LN group.

We further investigated the correlation between the serum levels of identified discriminatory metabolites and clinical disease activity in LN patients. Of different metabolites that were significantly changed in the sera of LN patients (Table 3), the lipid metabolites (including LDL/VLDL) and acetate showed statistically significant correlation with SLE disease activity index (SLEDAI). As shown in Fig. 5, the SLEDAI score has positive correlation with LDL/VLDL and lipids, whereas inverse correlation with acetate for LN group. We have also investigated the correlation between the discriminatory serum metabolites and SLE disease activity index (SLEDAI) for SLE group but no significant correlation was observed.

## Discussion

**Figure 6:**
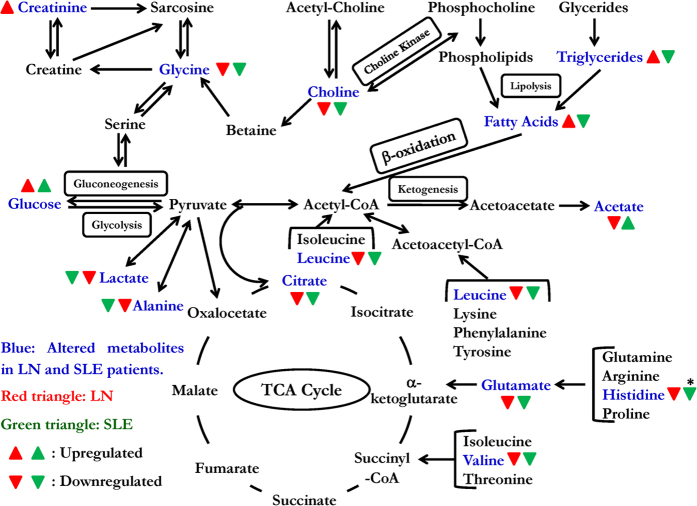
Schematic representation of altered metabolic pathways in LN and SLE patients. The altered metabolites are shown in blue color. The red and green triangles stand for LN and SLE patients, respectively. Up and down triangles denotes higher and lower levels of respected metabolites compared to healthy controls. (*Indicates not significant).

In the present study, we have demonstrated the potential of ^1^H NMR-based metabolomic approach for identifying LN patients from SLE patients based on their characteristic serum metabolite profiles. To the best of our knowledge, this is the first report on serum metabolic profiling for LN. The previous reports on SLE have shown that serum metabolome can result in the separation from HC^24^, and from rheumatoid arthritis (RA)^23^. However, these studies either did not categorize patients with nephritis or did not enrol LN as disease control and hence, did not demonstrate the ability of metabolic profiling to distinguish LN from SLE. The present study fills this lacuna and demonstrates that it is possible to separate LN patients from SLE patients and HC using both PCA and PLS-DA analysis applied to ^1^H-NMR spectra of human serum. The study revealed a wide range of differential metabolic signatures in SLE and LN patients. Significant metabolic disturbances were found in multiple cellular pathways, including glycolysis, amino acid metabolism and lipid metabolism. The metabolic network of potential biomarkers is presented in Fig. 6, which gives an overview of the metabolic pathways altered in SLE and LN conditions. The implications of these observed metabolic changes in the pathophysiology of LN have been discussed further.

An important feature accounting for the discrimination between patient cohorts and HC was the difference in relative levels of lipid and membrane metabolites. Compared to both SLE and HC, the LN patients showed significantly increased serum levels of VLDL/LDL and unsaturated lipids and decreased serum levels of acetate. Consistent with previous reports, hyperlipidemia (which might be related to impaired renal function) is the commonest manifestation of LN^26^, and represents a leading cause of cardiovascular complications and pre-mature mortality in LN patients^26^,^27^. Interestingly, the serum levels of lipid metabolites (including LDL/VLDL) showed positive and statistically significant correlation with SLEDAI score and this is also in line with previous observations that there is an increase in serum lipid levels with disease progression^26^. We have also observed an inverse correlation of SLEDAI score with acetate which is the end product of lipid metabolism and its decrease further reflects a disturbed lipid metabolism in LN patients. However, contrary to previous reports, the serum levels of VLDL/LDL and unsaturated lipids were found to be slightly decreased in SLE patients compared to HC. SLE patients are at increased risk of developing premature atherosclerosis and myocardial infarction associated with dyslipidemia such as raised LDL cholesterol, oxidized LDL (Ox-LDL) and low HDL^26^,^28^,^29^. B-cell depletion therapy has been shown to improve dyslipidemia implying the relation of dyslipidemia with disease activity^30^. The possible reason for decreased serum levels of LDL could be the excessive per-oxidation of circulatory LDL to Ox-LDL which is aberrantly involved in inflammatory processes through the formation of higher molecular weight complexes with distinct inflammatory mediators^31^,^32^. The NMR signal of these complexes are highly broadened and hidden in the spectral baseline rendering decreased NMR signal of LDL in sera of SLE patients. However, increased serum levels of acetate in SLE patients clearly indicate that the lipid metabolism is activated in SLE patients required to regulate the production of lipid and membrane metabolites.

Serum samples of SLE and LN patients contained significantly increased levels of N-acetyl glycoproteins (NAGs) and decreased levels of choline. NAGs are mainly acute phase proteins with anti-inflammatory properties and are expressed more during inflammation and immune responses^33^. Whereas, choline is an important intermediate of phospholipid metabolism, it is also a precursor in the synthesis of acetylcholine and phosphoryl-choline. Phosphoryl-choline is an essential component in membrane structure and therefore, decreased choline level in both SLE and LN patients might be related to the augmented utilization of phosphorylcholine for repairing the damaged cells/organelles under severe oxidative and systemic inflammatory condition. In concordance with serum choline levels, the present study also shows marked reduction in serum acetate levels in LN compared to SLE patients and HC suggesting acutely perturbed biosynthesis of acetyl-choline in LN.

Compared to HC, SLE/LN patients showed a significant increase in glucose accompanied by decrease in lactate levels, suggesting disturbed glucose metabolism with dampened aerobic glycolytic activity. Further, we have also observed decreased levels of citrate in the sera of SLE/LN patients, indicating impaired aerobic glycolysis and thus dampened oxidative phosphorylation and ATP production in these patients. The similar higher glucose levels and decreased lactate and citrate levels have also been found in the sera of SLE^24^ and Takayasu Arteritis (TA) patients^34^. Both SLE and TA are auto-immune diseases and clinically manifested with systemic inflammation which is known to trigger a hypercatabolic state, resulting in increased energy requirements and protein catabolism.

Our data also revealed decreased serum levels of several amino acids (such as glycine, alanine, valine, leucine, glutamate, proline and histidine) in both SLE and LN patients. Similar alterations have also been reported in autoimmune disorders associated with systemic inflammation and oxidative stress like SLE^23^,^24^, TA^34^ and RA^23^, suggesting aberrant amino acid catabolism and protein biosynthesis in these patients to regulate various biological functions such as gene transcription, cell cycle progression, inflammatory and autoimmune responses. Particularly, the lower serum levels of histidine –which is well-known for its anti-inflammatory and antioxidant properties– might be closely related to protein-energy wasting, inflammation and oxidative-stress^35^,^36^. Taken together, the elevated glucose levels in sera of SLE/LN patients and reduced levels of most of the glucogenic amino acids (such as glycine, alanine, glutamate, valine and histidine) and ketogenic amino acids (such as leucine), provided indications of a shift in energy production such as (a) profoundly dampened aerobic glycolysis and (b) utilization of metabolites other than glucose as an energy source, such as amino acids and ketone bodies. Our results also indicated an elevated serum level of creatinine in LN patients compared to SLE and HC, as expected with renal dysfunction. However, metabolomics revealed far more extensive differences between SLE and LN than could be learnt from a simple serum creatinine test.

The metabolic signatures in conjunction with biomarkers derived from proteomics and genomics studies have immense potential to predict disease course and follow treatment response which is a crucial pre-requisite to improve efficacy and decrease toxicity of the current treatment protocols. The present study provided proof of concept that NMR-based serum metabolomics approach has high sensitivity and specificity to discriminate LN from SLE cohorts. A potential metabolic hallmark discovered in this study apart from creatinine, is the elevated serum levels of LDL/VLDL lipoproteins (triglyceride and fatty acid) and decreased serum levels of acetate in LN compared to SLE patients. The obtained metabolic disturbances delineate some of the underlying biochemical processes and aid the understanding of the pathogenesis of this life-threatening complication of SLE. It is important to mark here that the current study was performed with a limited number of samples and the metabolic changes present in the sera of LN patients should be further validated on larger prospective patient cohorts. After validation, these differential metabolic signatures would be used in clinical settings to improve the diagnosis and clinical management of LN.

## Materials and Methods

### Ethical Approval

All experimental protocols of this study were approved by the Institutional Research Ethics Committee, SGPGIMS, Lucknow and informed consents were obtained from all patients and healthy volunteers before enrolment in the study. The methods used in this study were carried out in accordance with the approved protocols and guidelines.

### Subjects and participants

The serum samples used in this study were collected from SLE and LN patients attending the Department of Clinical Immunology at SGPGIMS, Lucknow, India. Serum samples were collected from twenty two patients with SLE (22 females, mean age 32.3 ± 11.9 years), forty patients with LN (37 females, 3 males, mean age 28.7 ± 10.1 years), and thirty healthy control subjects (25 females, 5 males, mean age 28.3 ± 5.87 years). Serum creatinine was measured using automated analyzer (Randof Diagnostics, UK, Model Imola). The normal range of serum creatinine is 0.5–1.6 mg/dl and of urea is 17–52 mg/dl. The demographics and clinical characteristics of SLE and LN patients are tabulated in Table 2.

### Sample collection and preparation for NMR spectroscopy

Venous blood samples were obtained from all subjects at early morning, after overnight fast to minimize the effect of dietary factors and inter-individual variations in metabolomics data^37^,^38^. Blood samples were kept in vacutainer tubes for 30 minutes at room temperature for clotting. Clotted blood samples were centrifuged at 16278 rcf (relative centrifugal force) for 10 minutes to separate out the supernatant-serum, which was then frozen and stored at a temperature of −80 °C, until the NMR measurements were performed. At the time of NMR measurement, serum samples were thawed at room temperature and mixed using a vortex mixer. Then, the aliquots of 250 μL of serum were transferred into 250 μL of saline buffer solution (in 100% D_2_O, NaCl 0.9%, 50 mM sodium phosphate buffer and pH 7.4) to minimize the variation in pH. The samples were centrifuged for 5 min at 6164 rcf and 450 μL of each sample supernatant was subsequently moved into a 5 mm NMR tube (Wilmad Glass, USA).

### NMR Measurements

The NMR experiments were performed at 298 K on Bruker Avance III 800 MHz NMR spectrometer (equipped with Cryoprobe) with a 5 mm broad-band inverse probe-head and Z-shielded gradient. The 450 μL of serum sample was filled in 5 mm NMR tube (Wilmad Glass, USA) and a sealed capillary tube containing the known concentration of 0.1 mM TSP (Sodium salt of 3-trimethylsilyl-(2,2,3,3-d4)-propionic acid) dissolved in deuterium oxide (D_2_O) was inserted separately both for the purpose of locking and chemical shift referencing. Deuterium oxide (D_2_O) and sodium salt of trimethylsilylpropionic acid-d_4_ (TSP) used for NMR spectroscopy were purchased from Sigma-Aldrich (Rhode Island, USA). One‐dimensional CPMG and diffusion edited ^1^H-NMR spectra were recorded on all the serum samples using the Carr–Purcell–Meiboom–Gill pulse sequence (cpmgpr1d, standard Bruker pulse program)^39^ and the bipolar pulse pair longitudinal eddy current delay (BPP-LED) sequence^40^, respectively. The parameters used for 1D CPMG pulse sequence were as follows: spectral sweep width: 12 ppm; data points: 32 K; flip angle of radiofrequnecy pulse: 90°; total relaxation delay (RD): 5 sec; T_2_ filtering was obtained with an echo time of 200 μs repeated 300 times, resulting in a total duration of effective echo time of 60 ms; number of scans: 128; window function: exponential and line broadening: 0.3 Hz. For diffusion edited ^1^H NMR pulse sequence, the square gradients of 70% of the maximum gradient strength (56 G/cm) and 2 ms duration (followed by a delay of 200 μs to allow for the decay of eddy currents) were used. Diffusion time of 120 ms was used to attenuate the signals from low molecular weight compounds without affecting the lipid signals. All the spectra or FIDs (free induction decays) were processed using Topspin-2.1 (Bruker NMR data Processing Software) using standard Fourier Transformation (FT) procedure following manual phase and baseline-correction. Prior to FT, each FID was zero-filled to 4096 data points and a sine–bell apodisation function was applied. 2D homonuclear ^1^H-^1^H total correlation spectroscopy (TOCSY) and ^1^H-^13^C heteronuclear single-quantum correlation spectroscopy (HSQC) spectra were acquired for selected samples to aid the spectral assignment. The details of various NMR parameters of these 2D homonuclear and heteronuclear experiments are given in Supplementary material Appendix I. Spectral resonances were identified and assigned as far as possible, by comparing them with the chemical shifts available in the database library of Chenomx Profiler (NMR Suite, v8.1, Chenomx Inc., Edmonton, Canada) and further validated (a) using the freely available software MetaboMiner^41^, (b) performing spiking experiments using standard chemicals (see Supplementary material, Figures S7 and S8) and (c) also using other existing databases and literature reports^42^,^43^,^44^,^45^.

### Pattern recognition analysis of NMR data

The multivariate data analysis was performed on the 1D ^1^H CPMG and diffusion edited NMR spectra for low molecular weight metabolites and lipids, respectively. To facilitate statistical analysis, the reduction of NMR data was done using Bruker AMIX software (Version 3.8.7, Bruker GmbH, Germany) in the chemical shift region δ0.5–8.5 ppm for CPMG and δ0.5–5.5 ppm for diffusion edited spcetra. The spectra were then binned into 0.01 ppm integrated spectral buckets. The chemical shift region δ4.7–5.2 ppm was excluded from the analysis to elminite the residual signal of water and distorted region due to water suppression. The lipid regions (0.7–0.9, 1.20–1.34, 1.52–1.56, 1.94–2.00, 2.19–2.23, 5.24–5.30 ppm) were also removed from the CPMG spectra to reveal the contribution of low molecular weight metabolites. The binned spectral data were obtained from AMIX after mean centering and normalization which was performed by dividing each data point by the sum of all data points present in the sample to compensate for the differences in concentration of metabolites among individual serum samples. The data were scaled using unit variance in which identical weight was given to all variables. The resulting data matrices were then exported into Microsoft Office Excel 2010 and used for multivariate analysis using Unscrambler X Software (Version 10.3, CAMO USA, Norway) and SIMCA (Version 14.0, Umetrics AB, Singapore). To be noted here is that the diffusion edited spectra contain signals mainly from lipid metabolites and these are not attenuated by overlapping signals of low molecular weight (MW) metabolites. Thus, DE spectra provide exquisite and reliable profiling of lipid metabolites. On the other hand, in CPMG spectra, the lipid signals may understate the statistical importance of low MW metabolites; therefore by excluding them from the analysis allows better comparison of low MW metabolites and surmounts their statistical importance as well. Therefore, lipid excluded CPMG spectral data has been complemented with diffusion edited spectra.

For determining the differences among HC and disease groups (SLE and LN), pair-wise multivariate analysis was performed for HC vs. LN, HC vs. SLE and LN vs. SLE groups. The obtained binned CPMG and BPP-LED NMR data matrices were first subjected to unsupervised principal component analysis (PCA) to examine inherent clustering and to identify outliers. To further demonstrate the differences between the different groups, supervised partial least square-discriminant analysis (PLS-DA) was employed to help identifying potential discriminatory metabolites. Model validation was done using repeated 7-fold internal cross validation. The reliability of the models were further rigorously validated by the permutation tests (n = 100) and CV-ANOVA (analysis of variance testing of cross-validated predictive residuals) tests. The receiver operating characteristic (ROC) analysis was obtained from Y predicted values to verify the robustness of PLS-DA models in discriminating the different cohorts. Area under the ROC curve (AUROC) was computed using the SPSS software (Version 11.2, IBM). The marker metabolites were identified from the loading plots (for PLS-DA) and the scores of variable importance on projection (VIPs).

Further, univariate analysis was performed by applying the independent samples T-test to several metabolites of interest (identified by the multivariate analysis) using SPSS software (Version 11.2, IBM). The correlations between the discriminatory serum metabolites and clinical disease activity index (SLEDAI score) were determined using the Pearson correlation coefficient. A 0.05 level of probability was used as the criterion for statistical significance.

## Additional Information

**How to cite this article**: Guleria, A. *et al*. NMR based serum metabolomics reveals a distinctive signature in patients with Lupus Nephritis. *Sci. Rep*. **6**, 35309; doi: 10.1038/srep35309 (2016).

## Supplementary Material

Supplementary Information

## Figures and Tables

**Table 1:** Goodness-of-fit of the PLS-DA models obtained from 1D CPMG and diffusion edited NMR-based analysis of serum samples.

Comparison	NMR spectra	R^2^X (cum)	R^2^Y (cum)	Q^2^ (cum)	CV-ANOVA, *p*-value	Number of latent variables
HC vs LN	1D CPMG	0.75	0.95	0.87	9.66 × 10^−18^	6
HC vs SLE	1D CPMG	0.75	0.94	0.86	1.66 × 10^−15^	4
LN vs SLE	1D CPMG	0.58	0.77	0.54	1.88 × 10^−6^	3
HC vs LN	1D Diffusion edited	0.86	0.84	0.68	6.42 × 10^−10^	5
HC vs SLE	1D Diffusion edited	0.67	0.81	0.61	2.26 × 10^−7^	3
LN vs SLE	1D Diffusion edited	0.65	0.73	0.57	2.23 × 10^−8^	2

**Table 2:** Demographics and Clinical characteristics of SLE, LN patients and Controls.

	SLE without Nephritis (SLE)	Lupus Nephritis (LN)	Healthy Control (HC)
**Number**	22	40	30
**Gender** (F/M)	22/0	37/3	25/5
**Age (years),** Mean ± SD	32.3 ± 11.9	28.7 ± 10.1	28.3 ± 5.87
**Duration of disease** (Years), Mean ± SD			
**Malar Rash** (Yes/No)	15/7	20/20	
**Oral ulcer** (Yes/No)	8/14	14/26	
**Discoid rash** (Yes/No)	4/18	4/36	
**Photosensitivity** (Yes/No)	16/6	21/19	
**Arthritis** (Yes/No)	9/13	13/27	
**Neurological** (Yes/No)	4/18	4/36	
**Serositis** (Yes/No)	0/22	3/37	
**Haematological** (Yes/No)	1/21	5/35	
**SLEDAI** (score), Mean ± SD	2.76 ± 3.06	6.4 ± 6.61	
**Serum Creatinine** (mg/dl), Mean ± SD	0.81 ± 0.22	1.03 ± 0.55	
**Urea** (mg/dl), Mean ± SD	31 ± 9.92	43.26 ± 33.88	
**Anti-dsDNA antibodies** n (%)	13 (60)	33 (82)	
**C3** (mg/dl), Mean ± SD	101.2 ± 46.6	92.20 ± 54.7	
**C4** (mg/dl), Mean ± SD	27.4 ± 20.9	21.3 ± 17.0	
**Drugs** n (%)			
Hydroxychloroquine	19 (86)	32 (80)	
Prednisolone (Steroid)	20 (91)	39 (98)	
Cyclophosphamide	0 (0)	4 (10)	
Azathioprine	3 (14)	16 (40)	
Mycophenolate	0 (0)	3 (8)	
Methotrexate	3 (14)	1 (2)	

**Table 3:** Key observed metabolic differences between HC and LN, between HC and SLE, and between LN and SLE.

Metabolites	Chemical shift	LN vs HC			SLE vs HC			LN vs SLE		
		Variation	VIP	p-value	Variation	VIP	p-value	Variation	VIP	p-value
Leu	0.95	↓	1.10	<0.001	↓	0.91	0.036	—	—	—
Val	1.02	↓	1.80	0.006	↓	1.22	0.046	—	—	—
Ala	1.45	↓	3.22	0.031	↓	2.17	0.035	—	—	—
Ace	1.90	↓	0.62	0.001	↑	0.42	<0.001	↓	1.44	<0.001
NAG	2.02	↑	2.65	<0.001	↑	0.99	<0.001	—	—	—
Glu	2.34	↓	1.42	<0.001	↓	0.94	0.001	—	—	—
Cit	2.51	↓	0.89	<0.001	↓	0.70	<0.001			
Chol	3.20	↓	2.72	0.032	↓	3.92	0.001	↑	4.53	0.002
Pro	3.33	↓	1.68	<0.001	↓	1.20	0.043	—	—	—
Gly	3.54	↓	3.33	0.01	↓	2.93	0.005	—	—	—
Lac	4.10	↓	8.98	<0.001	↓	7.89	<0.001	↑	3.13	<0.001
Glucose	3.23–3.90, 4.63, 5.21	↑	1.00–6.30	0.001	↑	1.00–6.33	0.001	↓	1.00–6.09	<0.001
His	7.03	↓	0.72	<0.001	—	—	—	—	—	—
L1\L2	0.78–0.90	↑	1.00–2.96	<0.001	↓	1.00–3.76	<0.001	↑	1.00–2.59	<0.001
L3\L4	1.20–1.29	↑	2.00–10.1	<0.001	↓	2.00–7.37	<0.001	↑	2.00–9.70	<0.001
L5	1.54	↑	0.78	<0.001	↓	0.58	<0.001	↑	0.81	<0.001
L6	1.99	↑	1.50	<0.001	↓	1.36	<0.001	↑	1.65	<0.001
L7	2.20	↑	1.06	<0.001	↓	0.90	<0.001	↑	1.10	<0.001
L8	2.71	↑	0.37	<0.001	↓	0.29	<0.001	↑	0.42	<0.001
L9	5.27	↑	1.40	<0.001	↓	1.19	<0.001	↑	1.32	<0.001

Chemical shift, variation, VIP score and p-values of the individual biomarkers are given. The statistical significance for various metabolites was determined by independent samples t-test. *p*-values less than 0.05 were considered as significant. The arrows ↑ and ↓ indicate increase and decrease of metabolite levels in the LN and SLE cohorts compared with healthy control group, in case of LN vs. HC and SLE vs. HC, respectively, and in LN group in case of LN vs. SLE analysis. Variable importance in the projection (VIP) was obtained from PLS-DA. The low molecular weight metabolite markers were obtained from the PLS-DA analysis of CPMG NMR spectra, while the lipids and lipoproteins were obtained from the PLS-DA analysis of diffusion edited NMR spectra.
