# Supplementary material for: NMR based serum metabolomics reveals a distinctive signature in patients with Lupus Nephritis
Source: Sci Rep. 2016 Oct 14;6:35309. doi: 10.1038/srep35309 (PMC5064370; doi:10.1038/srep35309)
Supplement: Supplementary Information [file srep35309-s1.pdf]

NMR based serum metabolomics reveals a distinctive signature in patients with Lupus Nephritis

Anupam Guleria[1*], Avadhesh Pratap[2], Durgesh Dubey[1,3], Atul Rawat[1,3], Smriti Chaurasia[2], Edavalath Sukesh[2], Sanat Phatak[2], Sajal Ajmani[2], Umesh Kumar[1], Chunni Lal Khetrapal[1], Paul Bacon[4], Ramnath Misra[2*] and Dinesh Kumar[1*]

[1]Centre of Biomedical Research, SGPGIMS Campus, Lucknow-226014, India

[2]Department of Immunology, SGPGIMS Campus, Lucknow-226014, India,

[3]Department of Biotechnology, Babasaheb Bhimrao Ambedkar University, Lucknow-226025, India

[4]Rheumatology Research Group, Division of Immunity and Infection, Birmingham University, UK

[*]Authors for Correspondence:

Dr. Anupam Guleria
(Assistant Professor)
Email: anuguleriaphy@gmail.com
Centre of Biomedical Research (CBMR),
SGPGIMS Campus, Lucknow-226014, Uttar Pradesh, India
Mobile: +91-9044866342

Dr. Dinesh Kumar
(Assistant Professor)
Email: dineshcbmr@gmail.com
Centre of Biomedical Research (CBMR),
SGPGIMS Campus, Lucknow-226014, Uttar Pradesh, India
Mobile: +91-8953261506

Prof. R Misra
Email: rnmisra2000@gmail.com
Former Dean and Professor and Head
Clinical Immunology,
SGPGIMS, Lucknow-226014, Uttar Pradesh, India
Phone +915222494284

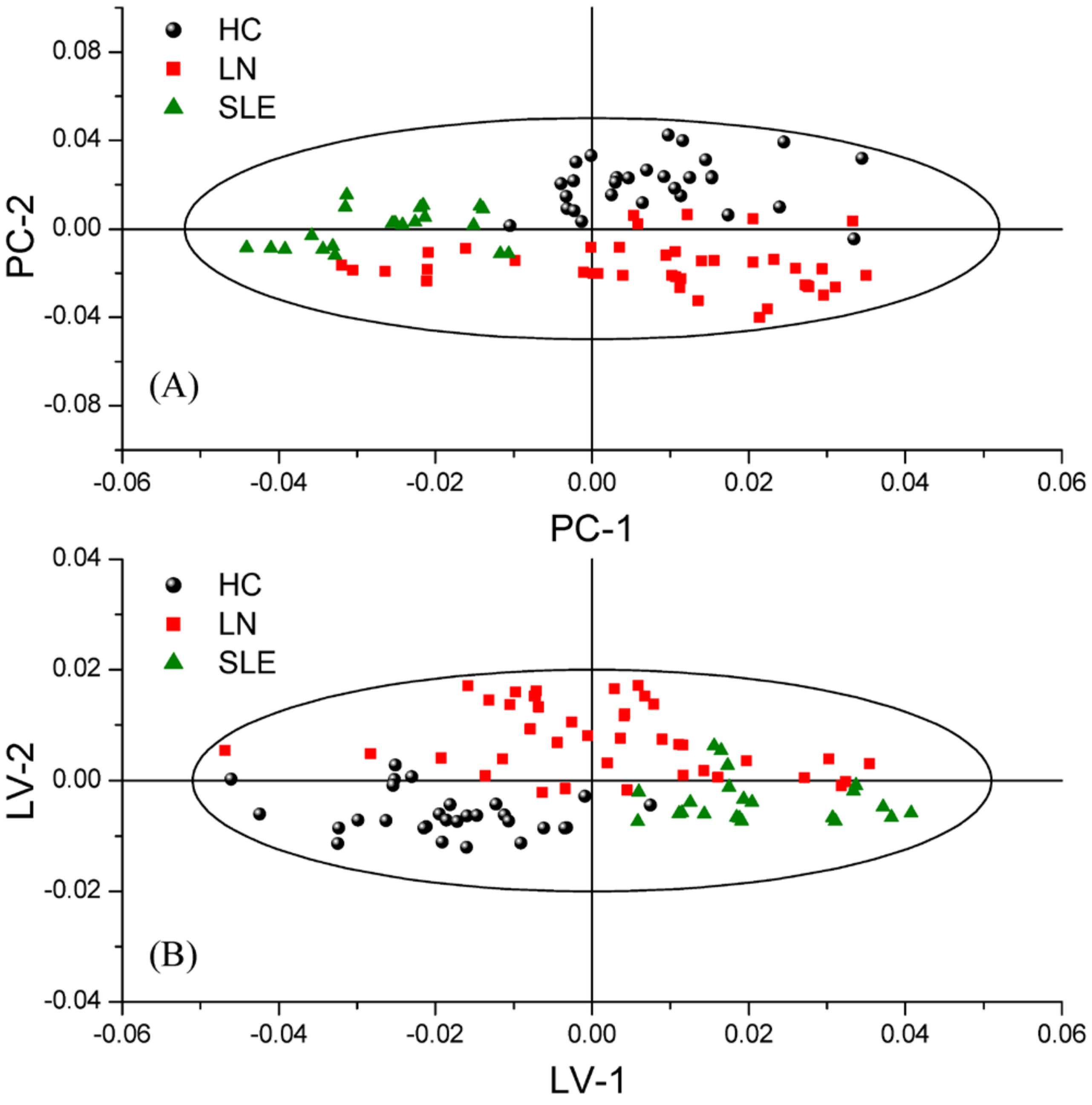

Figure S1: Two dimensional PCA and PLS-DA score plots showing a clear trend of group clustering and discrimination between the three cohorts i.e. healthy controls, SLE and LN patients.

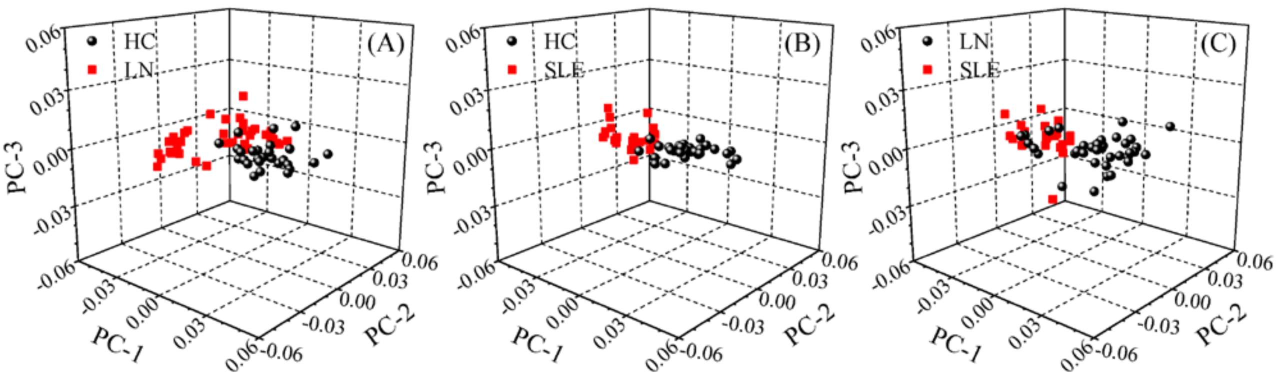

Figure S2: PCA score plots derived from 1D CPMG ^{1}H NMR spectra of serum samples between, A) HC and LN, B) HC and SLE, and C) LN and SLE.

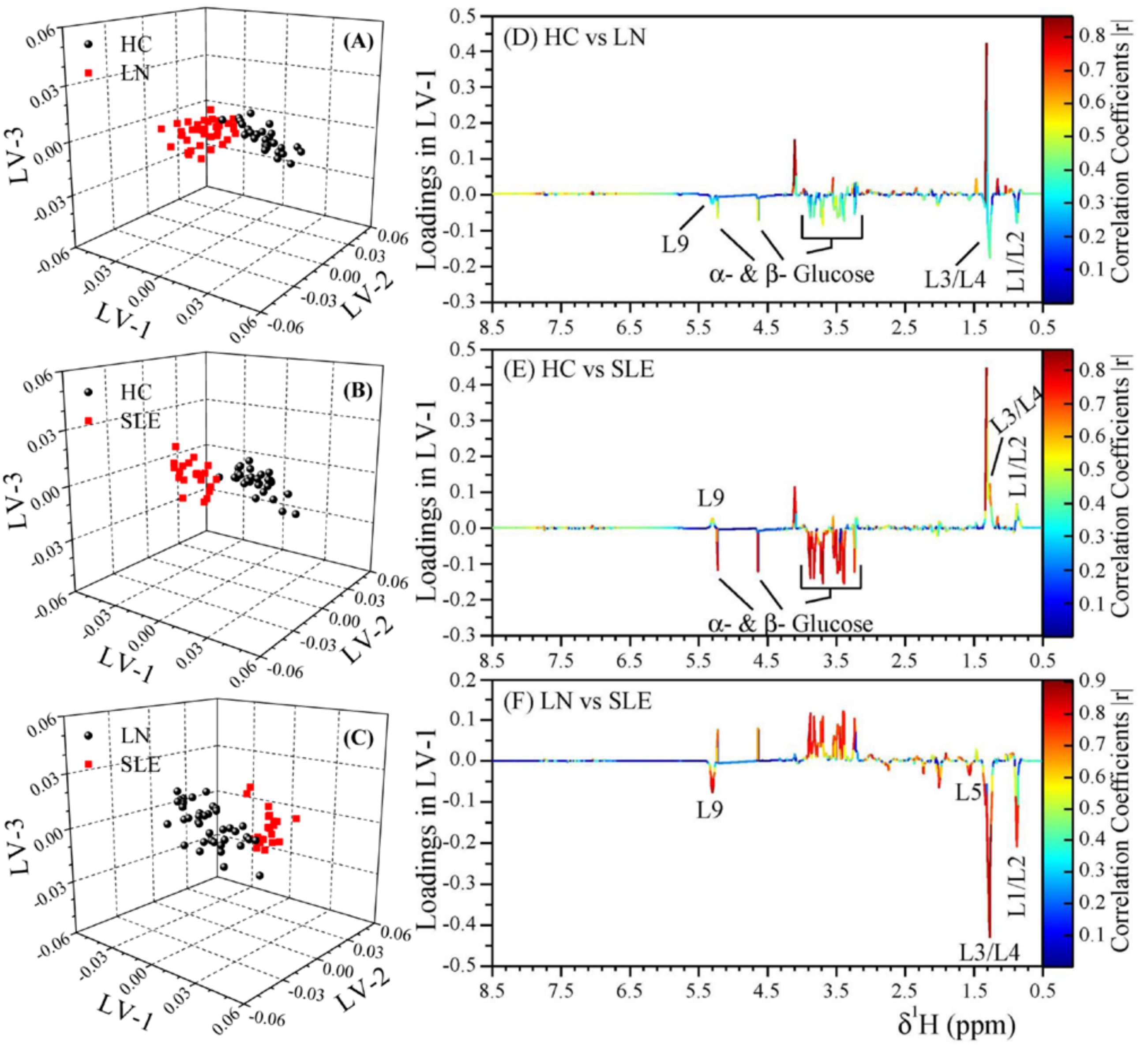

Figure S3: PLS-DA score plots derived from 1D CPMG ^{1}H NMR spectra of serum samples (including the lipid regions), between, A) HC & LN, B) HC & SLE, and C) LN & SLE. (D, E & F) shows the color coded coefficient loading plot corresponding to the PLS-DA analysis shown in (A, B & C), respectively. Loading plots clearly demonstrates the variations in the lipoproteins/lipids signals are similar to as observed from the diffusion edited results.

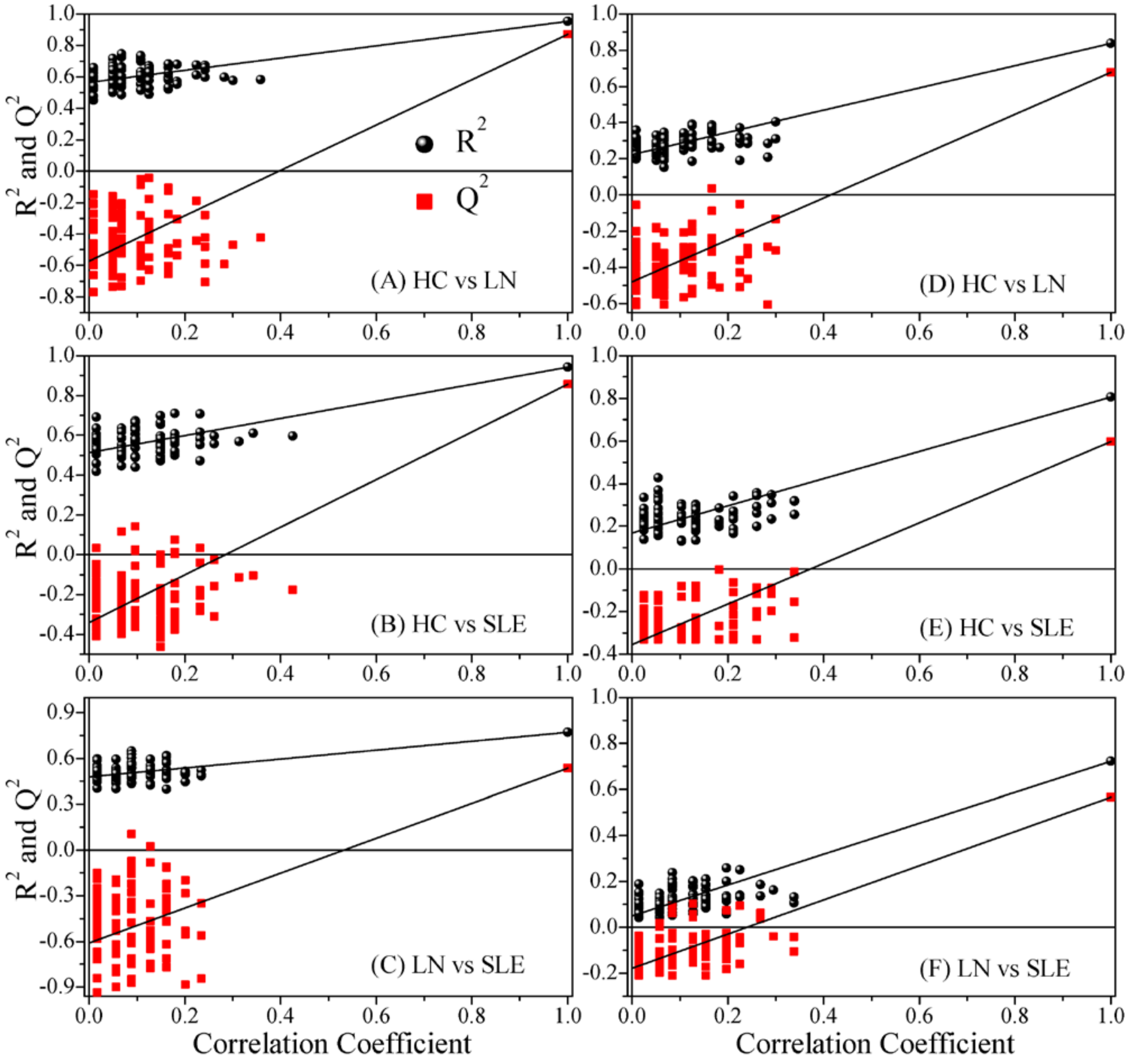

Figure S4: Permutation analysis using 100 different model permutations of PLS-DA models derived from CPMG spectra (A, B & C) and diffusion edited spectra (D, E & F) of HC vs LN, HC vs SLE and LN vs SLE groups, respectively. The goodness of fit (R^2) and predictive capability (Q^2) of the original models are indicated on the far right and remain higher than those of the 100 permuted models to the left in all cases.

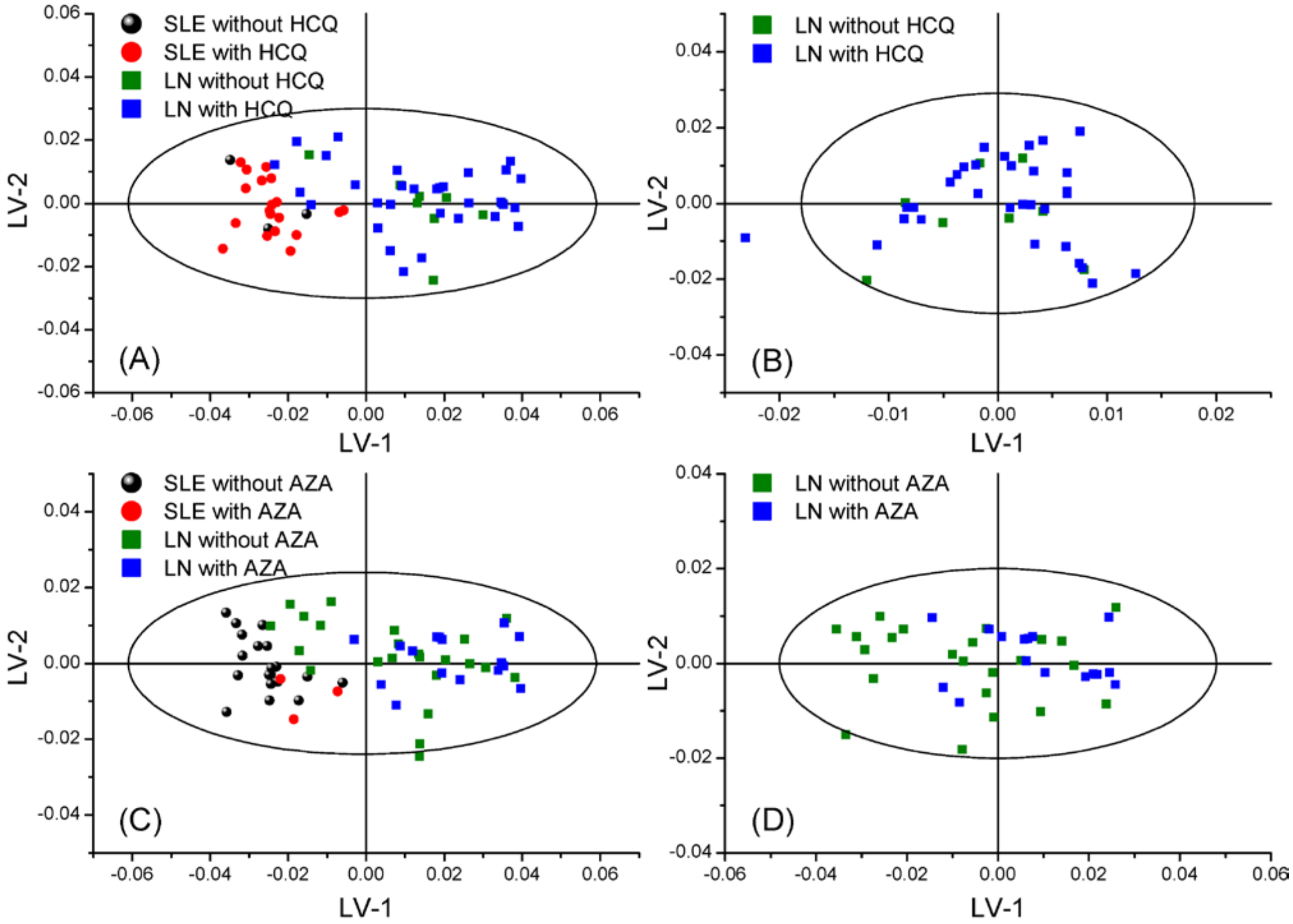

Figure S5: PLS-DA score plot generated from ^{1}H NMR spectra of serum samples obtained from (A) SLE and LN patients receiving hydroxychloroquine (HCQ) and not receiving HCQ, (B) LN patients receiving HCQ and not receiving HCQ, (C) SLE and LN patients with azathioprine (AZA) and without AZA, and (D) LN patients with AZA and without AZA.

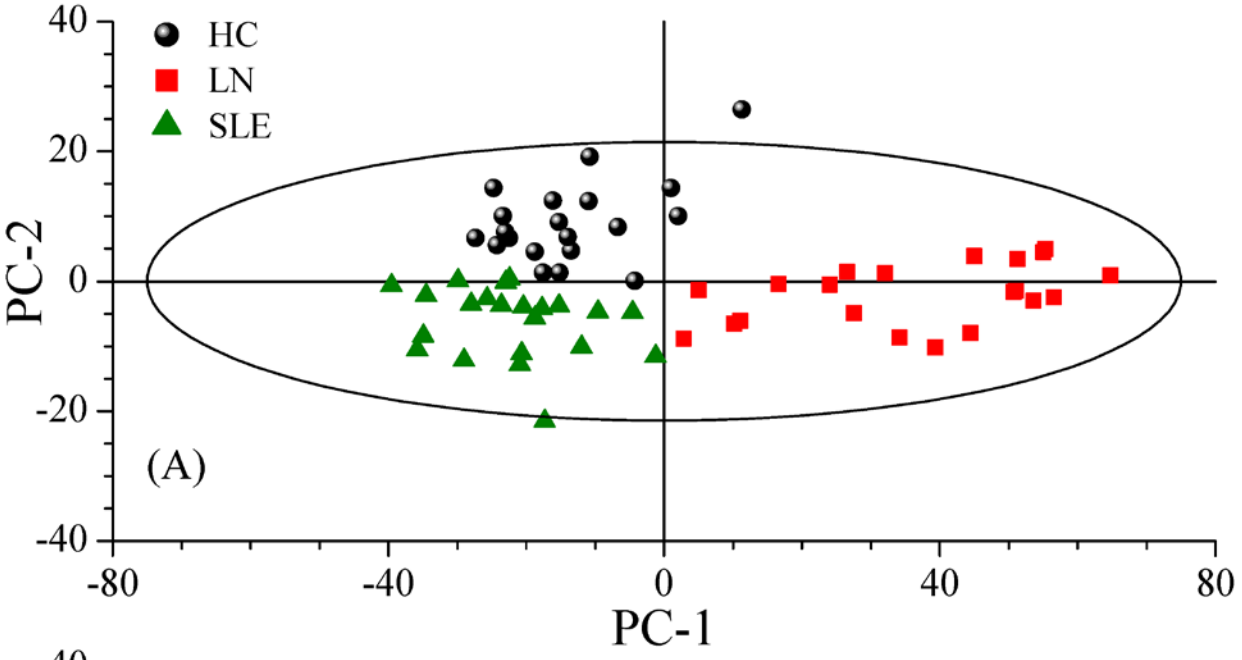

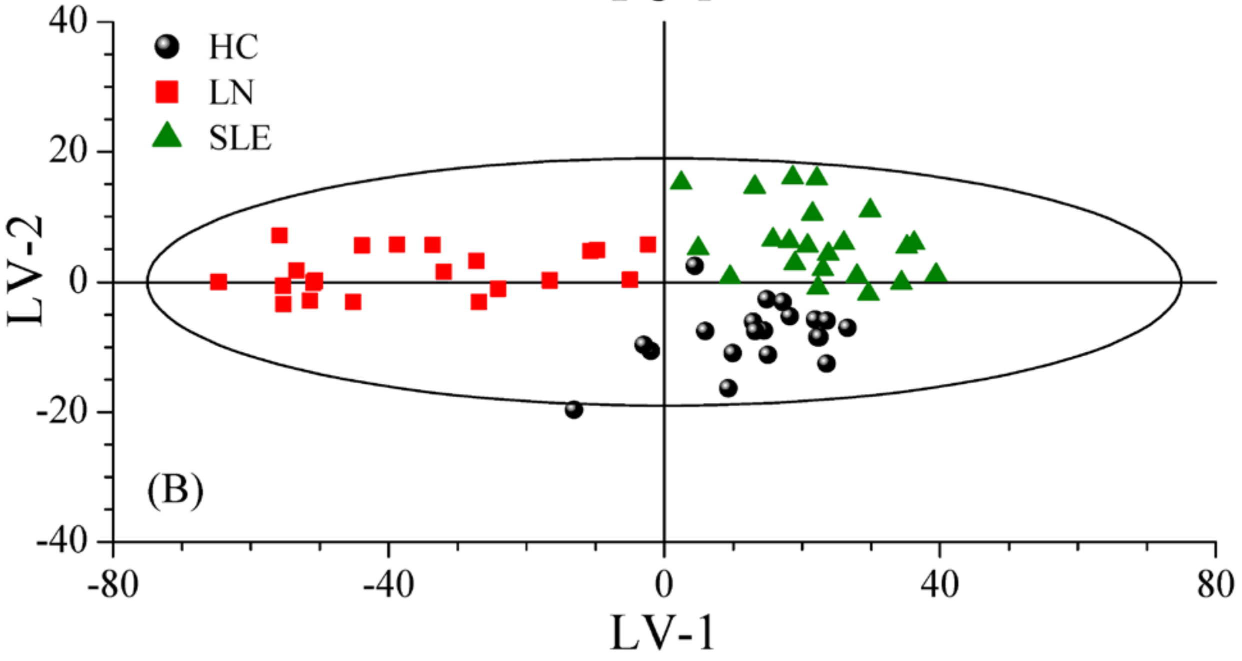

Figure S6: PCA and PLS-DA score plots generated using the quantified metabolite data showing the clear separation between the three groups (20 samples from each group).

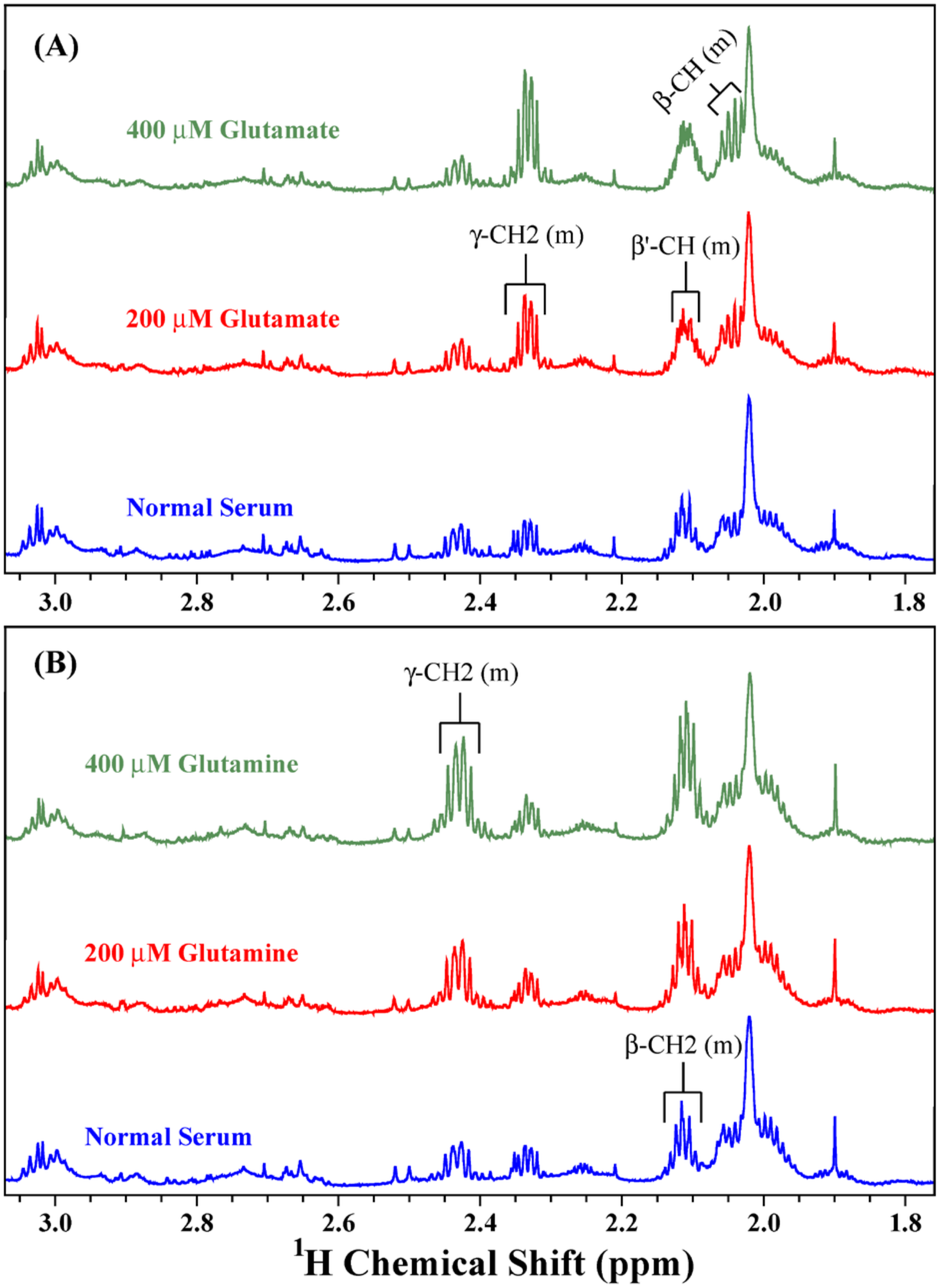

Figure S7: The assignment of peaks corresponding to serum metabolite **(A)** L-glutamate and **(B)** L-glutamine confirmed using spiking method following sequential addition of L-glutamate and L-glutamine solution, respectively, prepared in saline D_2O (all purchased from Sigma-Aldrich) into control serum sample.

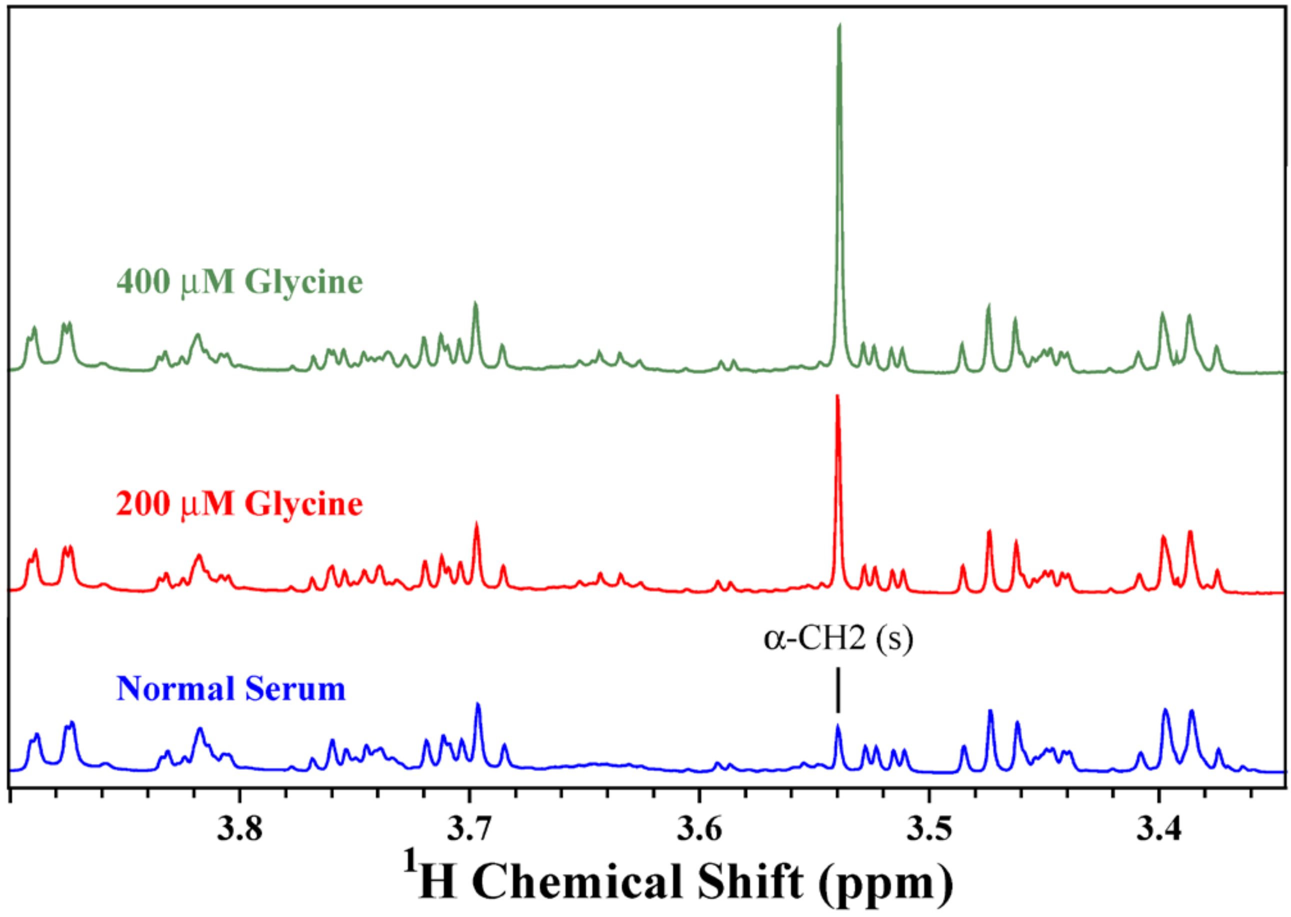

Figure S8: The assignment of peaks corresponding to serum metabolite glycine confirmed using spiking method following sequential addition of glycine solution prepared in saline D_2O (all purchased from Sigma-Aldrich) into control serum sample.

Appendix I:

Unambiguous assignments of various metabolites were obtained using two-dimensional TOCSY (total proton-proton correlation spectroscopy) and HSQC (heteronuclear single quantum correlation) spectra (acquired on three-to-four serum samples) and spiking experiments using standard chemicals. Two-dimensional ^{1}H-^{1}H TOCSY (dipsi2esgpph) and sensitivity enhanced ^{1}H–^{13}C HSQC (hsqcetgp) spectra were acquired in phase sensitive mode using time proportional phase incrementation (TPPI). 2D TOCSY spectrum was recorded using 2048 data points along direct dimension (F_2) and 512 increments along indirect dimension (F_1) with 16 transients per increment and a spectral width of 12 ppm in both dimensions. The FIDs were weighted using a sine–bell-squared function in both dimensions and zero filled to 2048 and 4096 data points, respectively, in the F_1 and F_2 dimensions prior to FT. The RD between successive pulse cycles was 3.0 sec for TOCSY experiment (with mixing time of 80 ms). Spin-lock was achieved by a DIPSI2 pulse sequence train during the TOCSY mixing time. 2D HSQC spectrum was recorded with inverse ^{13}C detection and using ^{13}C decoupling during acquisition using GARP-1. A RD of 2.0 sec was used between successive pulse sequence cycles and a refocusing delay equal to $1/(4{*}^{1}J_{C\text{–}H}$ =1.75 ms) was employed. 512 x 128 complex t_2 (^{1}H) and t_1(^{13}C) data points with 96 scans per increment were acquired with spectral widths of 12 and 165 ppm, respectively, in the ^{1}H and ^{13}C dimensions. The FIDs were weighted using a sine-bell-squared function in both dimensions and zero filled to 1024 and 2048 data points, respectively, in the F_1 and F_2 dimensions prior to FT. After FT, the final spectrum was manually phase corrected and ^{1}H and ^{13}C dimensions were referenced to lactate methyl protons and carbon, respectively, at 1.31 and 22.5 ppm.

Table S1: Evaluation of statistical significance of quantified metabolites as observed in the ^{1}H NMR spectra of HC, LN and SLE patients (randomly selected 20 samples in each group). Relative integrals of metabolite resonances of interest were used for statistical comparison among groups. *P*-values less than 0.05 were considered as significant.

S. No.	Metabolites	HC (n=20) Median (Range)	LN (n=20) Median (Range)	SLE (n=20) Median (Range)	p-value HC vs LN	p-value HC vs SLE	p-value LN vs SLE
1.	Valine	1.56 (1.07-2.11)	1.37 (0.99-1.73)	1.37 (0.99-1.72)	0.006	0.046	0.451
2.	Alanine	3.93 (2.69-5.34)	3.20 (1.85-4.5)	3.11 (1.73-4.2)	0.031	0.035	0.706
3.	Acetate	0.21 (0.12-0.54)	0.14 (0.03-0.60)	0.46 (0.28-0.68)	0.019	<0.001	<0.001
4.	NAG	2.62 (2.37-4.34)	4.33 (3.20-7.03)	3.98 (2.64-6.17)	<0.001	<0.001	0.301
5.	Glutamate	1.45 (1.05-2.05)	0.92 (0.53-1.44)	1.03 (0.73-1.63)	<0.001	0.001	0.059
6.	Citrate	0.34 (0.29-0.42)	0.24 (0.16-0.29)	0.21 (0.12-0.30)	<0.001	<0.001	0.115
7.	Creatinine	0.32 (0.21-0.50)	0.39 (0.29-1.81)	0.31 (0.19-0.67)	0.036	0.345	0.048
8.	Choline	1.07 (0.62-3.20)	1.00 (0.37-1.36)	0.61 (0.09-0.96)	0.032	0.001	0.002
9.	Glycine	1.56 (1.10-2.35)	1.26 (0.82-1.76)	1.22 (0.67-1.66)	0.01	0.005	0.443
10.	Histidine	0.19 (0.16-0.21)	0.16 (0.12-0.18)	0.16 (0.12-0.23)	<0.001	0.200	0.099
11.	L1\L2	16.54 (13.5-24.9)	31.9 (12.8-52.72)	12.97 (4.65-17.9)	<0.001	<0.001	<0.001
12.	L3\L4	10.7 (7.43-14.4)	25.3 (6.83-52.57)	7.1 (3.16-11.2)	<0.001	<0.001	<0.001
13.	L5	0.8 (0.34-1.35)	2.11 (0.21-4.61)	0.33 (0.1-1.00)	<0.001	0.015	<0.001
14.	L9	4.9 (2.72-7.32)	10.41 (2.2-18.9)	2.81 (0.75-5.2)	<0.001	<0.001	<0.001

Table S2: AUC of ROC of the individual biomarkers obtained from the PLS-DA models between LN vs HC, SLE vs HC and LN vs SLE.

No.	Metabolites	Chemical shift	LN vs HC	SLE vs HC	LN vs SLE
1.	Leu	0.95	0.82	0.82	-
2.	Val	1.02	0.93	0.94	-
3.	Ala	1.45	0.87	0.87	-
4.	Ace	1.90	0.81	0.94	1.000
5.	NAG	2.02	0.95	0.92	-
6.	Glu	2.34	0.98	0.94	-
7.	Cit	2.51	0.99	0.98	
8.	Chol	3.20	0.76	0.93	0.82
9.	Pro	3.33	0.82	0.76	-
10.	Gly	3.54	0.89	0.93	-
11.	Lac	4.10	0.96	0.95	0.75
12.	Glucose	3.23-3.90, 4.63, 5.21	0.80-0.99	0.80-1.00	0.75-0.92
13.	His	7.03	0.85	-	-
14.	L1\L2	0.78-0.90	0.94	0.92	0.98
15.	L3\L4	1.20-1.29	0.98	0.98	0.99
16.	L5	1.54	0.87	0.87	0.96
17.	L6	1.98	0.92	0.86	0.96
18.	L7	2.20	0.88	0.87	0.96
19.	L8	2.71	0.83	0.83	0.93
20.	L9	5.27	0.96	0.91	0.98

Note: "-" means that the AUROC is less than the 0.7.
